# Bradycardia Risk Associated with Intravenous Dexmedetomidine: A Retrospective Study of Risk Factors and Clinical Outcomes in Critically Ill Patients (BRAID)

**DOI:** 10.21203/rs.3.rs-7547505/v1

**Published:** 2025-09-23

**Authors:** Albahi Malik, Navya Ramesh, Craig M. Coopersmith, Jonathan Sevransky, Sivasubramanium V. Bhavani

**Affiliations:** Emory University School of Medicine; Emory University School of Medicine; Emory University; Emory University School of Medicine; Emory University School of Medicine

**Keywords:** Dexmedetomidine, Bradycardia, Critical care, Risk factors, Mortality, Sedation, Intensive care

## Abstract

**Background:**

Dexmedetomidine is a selective α2-adrenergic agonist commonly used for sedation in intensive care units (ICUs). While effective, it is associated with cardiovascular side effects, particularly bradycardia that may limit its use. Concerns about bradycardia-related complications often influence clinical decision-making regarding dexmedetomidine dosing and monitoring, yet the true clinical significance of this side effect remains unclear. This study aimed to identify risk factors for dexmedetomidineassociated bradycardia and evaluate its impact on clinical outcomes.

**Methods:**

We conducted a retrospective cohort study involving adult patients who were mechanically ventilated and received dexmedetomidine for sedation across 12 ICUs in 4 hospitals under one hospital system. Bradycardia was defined as a heart rate below 60 beats per minute within 24 hours after dexmedetomidine administration. Multivariable logistic regression was employed to identify independent risk factors and assess the association between bradycardia and clinical outcomes, including inpatient mortality.

**Results:**

Among 5,106 patients, 1,143 (22.4%) developed bradycardia. Independent risk factors for bradycardia included moderate/severe liver disease (OR 1.87, 95% CI 1.42–2.47), dementia (OR 1.67, 95% CI 1.21–2.31), cerebrovascular disease (OR 1.32, 95% CI 1.15–1.51), and cancer (OR 1.25, 95% CI 1.06–1.47). Lower risk of bradycardia included congestive heart failure (OR 0.71, 95% CI 0.63–0.80) and myocardial infarction (OR 0.73, 95% CI 0.63–0.85). Crude mortality rates were similar between groups (11.4% vs. 10.2%, p = 0.297), and after adjustment for age and illness severity, bradycardia was not independently associated with hospital mortality (adjusted OR 0.99, 95% CI 0.79–1.23, p = 0.915). Patients with bradycardia had shorter ICU length of stay compared to those without bradycardia (10.4 ± 10.1 vs. 10.8 ± 10.6 days, p = 0.012). A sensitivity analysis adjusting for minimum heart rate before dexmedetomidine initiation and using a stricter bradycardia definition of heart rate less than 50 bpm, resulted in similar findings.

**Conclusions:**

Dexmedetomidine-associated bradycardia occurs in approximately one-quarter of ICU patients at out institution. Specific comorbidities significantly influence the risk of developing bradycardia, but dexmedetomidine-associated bradycardia itself may not associated with worse outcomes.

## Introduction

Sedation management is a cornerstone of care for critically ill patients in intensive care units (ICUs), particularly for those requiring mechanical ventilation or undergoing invasive procedures.^1^ Over the past two decades, dexmedetomidine, a highly selective α2 adrenergic receptor agonist, has emerged as a valuable agent in the sedation armamentarium^2^ that provides sedation, anxiolysis, and modest analgesic effects without significant respiratory depression.^3^ These properties make it attractive for use in critically ill patients, especially during weaning from mechanical ventilation or when respiratory depression must be avoided, and it facilitates a more interactive level of sedation where patients can be easily aroused and communicate, potentially reducing delirium and improving overall ICU outcomes.^4,5^

Despite these advantages, dexmedetomidine is associated with notable cardiovascular side effects, primarily bradycardia and hypotension. These effects stem from its sympatholytic properties and central reduction of norepinephrine release.^6^ Bradycardia, defined as a heart rate below 60 beats per minute, is reported in clinical trials with varying frequencies ranging from 4–42% of patients receiving dexmedetomidine.^7,8^ This wide variation suggests that patient-specific factors may significantly influence the risk of developing this adverse effect. The clinical significance of dexmedetomidine-associated bradycardia remains incompletely understood. While often transient and well-tolerated, severe or prolonged bradycardia can lead to hemodynamic instability, reduced cardiac output, and potentially contribute to adverse outcomes in critically ill patients with limited physiological reserve.^9^

The present study aimed to address these knowledge gaps by analyzing a large cohort of critically ill patients receiving dexmedetomidine. Our primary objectives were to: (1) determine the incidence of bradycardia in ICU patients receiving dexmedetomidine; (2) identify independent risk factors for developing bradycardia; and (3) evaluate the association between bradycardia and clinical outcomes including mortality and length of stay. We hypothesized that specific patient characteristics and comorbidities would be associated with increased risk of bradycardia, and that the development of bradycardia would be associated with worse clinical outcomes.

## Methods

### Study Design and Population

We conducted a retrospective cohort study using data from adult patients (≥ 18 years) who received dexmedetomidine for sedation in medical, surgical, cardiac and neurological intensive care units at four Emory University hospitals between January 2011 and December 2022. The study was approved by the Institutional Review Board of Emory University (STUDY00007766). The requirement for informed consent was waived by the Institutional Review Board due to the retrospective nature of the study, the minimal risk to the participants, and the impracticability of obtaining consent from all patients over the 12-year study period. The research could not be practicably carried out without the waiver, and the waiver does not adversely affect the rights and welfare of the subjects.

#### Data Collection

Demographic data collected included age, sex, race, and body mass index (BMI). Clinical data included admission diagnosis, comorbidities, Sequential Organ Failure Assessment (SOFA) score, mechanical ventilation status, and disposition. Comorbidities were identified using established diagnostic codes and included myocardial infarction, congestive heart failure, peripheral vascular disease, cerebrovascular disease, dementia, chronic pulmonary disease, diabetes mellitus, Hypertension, renal disease, cancer, moderate/severe liver disease.

Heart rate data were collected throughout the dexmedetomidine infusion period. Bradycardia was defined as a recorded heart rate below 60 beats per minute at any point during dexmedetomidine administration within the first 24 hours. The primary outcome of interest was the patients risk factors associated with developing bradycardia. Secondary outcomes included in-hospital mortality and ICU length of stay (LOS).

### Statistical Analysis

Descriptive statistics were used to summarize patient characteristics. Continuous variables were reported as means with standard deviations, and categorical variables as counts with percentages. Comparisons between patients with and without bradycardia were performed using Student’s t-test for continuous variables and chi-square test for categorical variables.

To identify independent risk factors for bradycardia, we performed multivariable logistic regression analysis. The model was adjusted for age, SOFA score, BMI and minimum heart rate before receiving dexmedetomidine. Results were reported as odds ratios (ORs) with 95% confidence intervals (CIs).

Sensitivity analyses were conducted to evaluate predictors of bradycardia under varying definitions. This included assessing early bradycardia, defined as occurring within the first 6 hours of dexmedetomidine administration. Further sensitivity analysis focused on patients with a heart rate less than 50 bpm for the first 24 hours while on dexmedetomidine. All statistical analyses were performed using R version 4.5.0 (R Foundation for Statistical Computing, Vienna, Austria). A two-sided p-value of < 0.05 was considered statistically significant.

## Results

### Patient Characteristics

A total of 5,106 mechanically ventilated patients who received dexmedetomidine during the study period were included in the analysis. The mean age of the study population was 59.5 (15.4) with 56.9% male patients.

**Figure 1:**
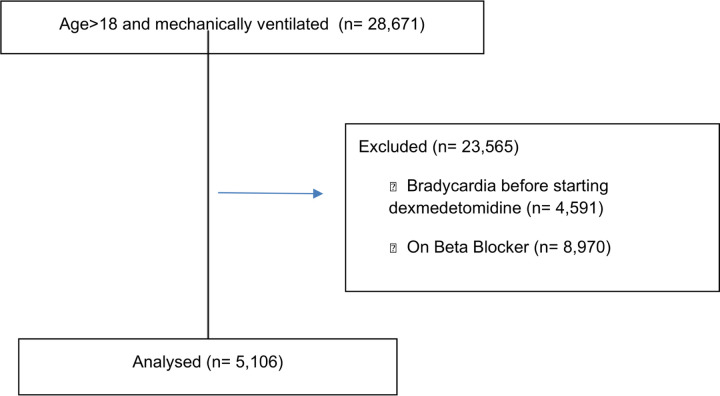
CONSORT Flow Diagram showing patient selection process.

The initial cohort included patients above 18 years who are mechanically ventilated (28,671) of whom 23,565 were excluded to having bradycardia before starting dexmedetomidine, being on heart rate reducing medication before start dexmedetomidine, or were on paralytics. After applying exclusion criteria, the final cohort comprised 5,106 patients (Fig. 1).

#### CONSORT Flow Diagram

Baseline characteristics of the study population are presented in Table 1 (end of document), stratified by the outcome of bradycardia. Patients who developed bradycardia were significantly older (61.6 ± 15.2 vs. 58.8 ± 15.5 years, p < 0.001) and there were a lower proportion of males (53.6% vs. 57.9%, p = 0.012). The bradycardia group showed higher rates of several comorbidities, including dementia (7.6% vs. 4.3%, p < 0.001), chronic pulmonary disease (40.3% vs. 36.0%, p = 0.009), and moderate/severe liver disease (10.1% vs. 7.1%, p = 0.002). Conversely, patients with bradycardia had significantly lower rates of peripheral vascular disease (25.1% vs. 30.9%, p < 0.001) and diabetes mellitus (16.4% vs. 20.6%, p = 0.002). Rates of myocardial infarction (18.0% vs. 19.1%, p = 0.423), congestive heart failure (50.4% vs. 53.2%, p = 0.100), and cerebrovascular disease (34.3% vs. 31.5%, p = 0.081) were not significantly different between groups. The bradycardia group had higher illness severity as measured by SOFA score (4.7 ± 3.1 vs. 4.4 ± 3.1, p < 0.001), while BMI was similar between groups (29.9 ± 10.3 vs. 29.3 ± 8.8, p = 0.595).

### Risk Factors for Bradycardia

**Figure 2:**
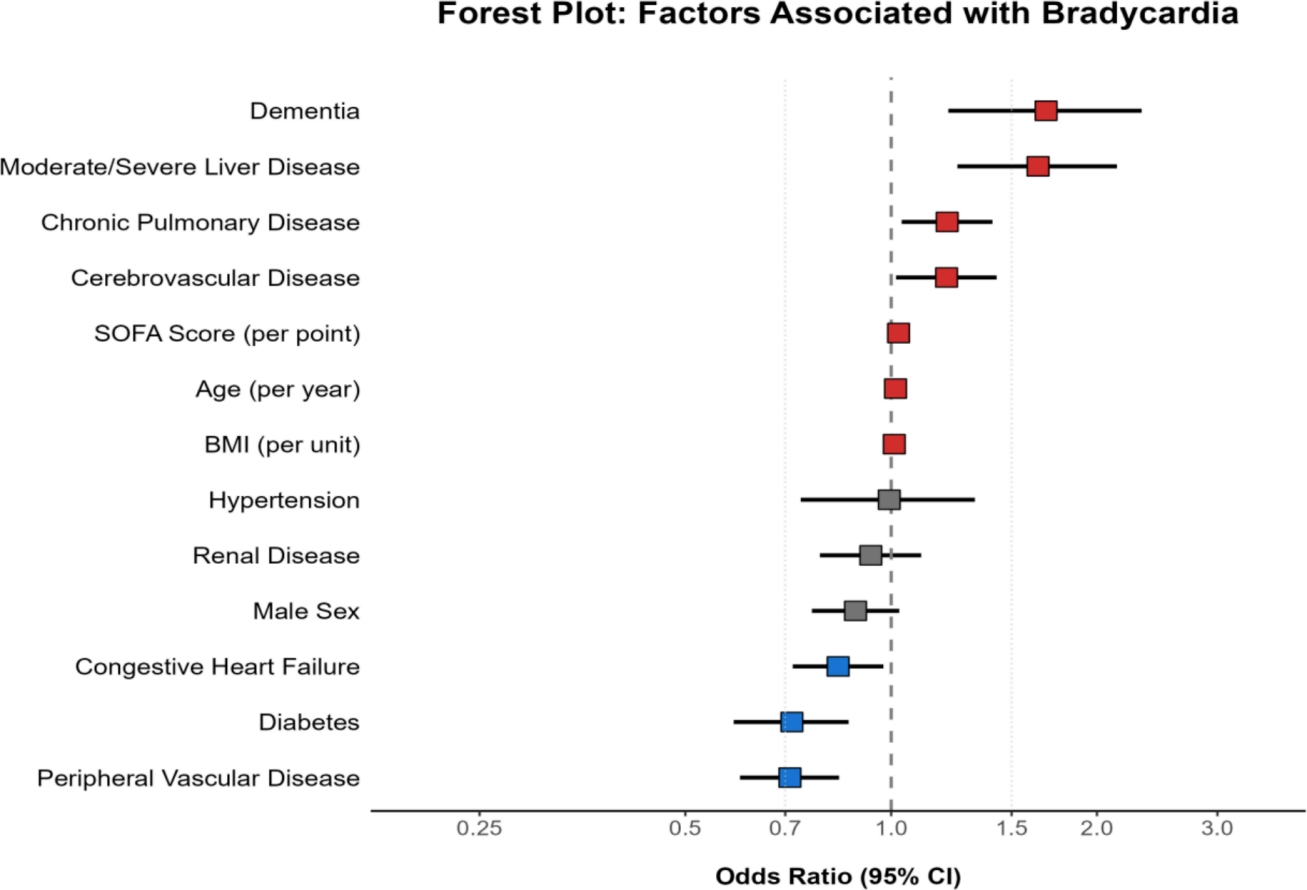
Forest plot of factors associated with bradycardia

In multivariable analysis adjusted for age, sex, BMI, and SOFA score (Fig. 2) (Table S1), several comorbidities were independently associated with bradycardia risk. Risk factors for bradycardia included cerebrovascular disease (OR 1.22, 95% CI 1.03–1.46, p = 0.025), dementia (OR 1.52, 95% CI 1.09–2.12, p = 0.014), chronic pulmonary disease (OR 1.26, 95% CI 1.08–1.48, p = 0.004), and moderate to severe liver disease (OR 1.44, 95% CI 1.09–1.90, p = 0.010). Lower risk included congestive heart failure (OR 0.78, 95% CI 0.66–0.91, p = 0.002), peripheral vascular disease (OR 0.70, 95% CI 0.59–0.84, p < 0.001), and diabetes mellitus (OR 0.75, 95% CI 0.62–0.92, p = 0.005).

Among the adjustment variables, all three were significantly associated with bradycardia risk: age (OR 1.01 per year, 95% CI 1.00–1.02, p = 0.023), BMI (OR 1.02 per kg/m^2^, 95% CI 1.01–1.03, p = 0.002), and SOFA score (OR 1.04 per point, 95% CI 1.01–1.06, p = 0.004).

A sensitivity analysis that additionally adjusted for minimum heart rate before dexmedetomidine initiation was conducted (Figure S1). Baseline heart rate was included as a continuous variable in the multivariable logistic regression model, along with age, BMI, and SOFA score. This approach allowed us to account for each patient’s pre-existing heart rate as a potential confounder in the relationship between comorbidities and bradycardia risk. This analysis yielded notably similar results, with dementia (OR 1.48, 95% CI 1.06–2.06), chronic pulmonary disease (OR 1.24, 95% CI 1.06–1.45), and moderate/severe liver disease (OR 1.42, 95% CI 1.08–1.87) maintaining a higher risk of developing bradycardia. Cerebrovascular disease (OR 1.18, 95% CI 0.99–1.41) also showed a trend towards higher risk but was not statistically significant in this adjusted model. Congestive heart failure, peripheral vascular disease, and diabetes mellitus maintained their significant lower risk of bradycardia.

Further sensitivity analysis was performed on patients with a stricter definition of bradycardia, specifically a heart rate less than 50 bpm for the first 24 hours while on dexmedetomidine. In this analysis, moderate/severe liver disease (OR 1.52, 95% CI 1.09–2.21) still showed a higher incidence of bradycardia, while other comorbidities were statistically insignificant, likely due to a lower number of events [Figure S2, Table S2].

### Clinical Outcomes

#### Association Between Bradycardia and Hospital Mortality

Crude mortality rates were similar between patients with and without bradycardia (11.4% vs. 10.2%, p = 0.297). In multivariable logistic regression analysis adjusted for age, sex, BMI and Sequential Organ Failure Assessment (SOFA) score, bradycardia was not independently associated with hospital mortality (adjusted OR 1.03, 95% CI 0.80–1.32, p = 0.8). As expected, both age (adjusted OR 1.03 per year, 95% CI 1.02–1.03, p < 0.001) and SOFA score (adjusted OR 1.32 per point, 95% CI 1.28–1.35, p < 0.001) remained significant independent predictors of hospital mortality, with each year of age increasing mortality odds by 3% and each SOFA point increase associated with a 32% increase in mortality odds.

#### Length of Stay

Patients with bradycardia had significantly shorter ICU length of stay compared to those without bradycardia (10.4 ± 10.1 vs. 10.8 ± 10.6 days, p = 0.012).

##### Association Between Bradycardia and Hospital Mortality (Multivariable Logistic Regression) Adjusted for Age, Sex, BMI, and SOFA Score

**Table:** 

Variable	OR	95% CI	p-value
**Bradycardia**	1.03	0.80, 1.32	0.8
**Age**	1.03	1.02, 1.03	<0.001
**Sex**	0.94	0.76, 1.16	0.6
**BMI**	1.00	0.99, 1.02	0.5
**SOFA Score**	1.33	1.29, 1.37	<0.001

Abbreviations: CI = Confidence Interval, OR = Odds Ratio

## Discussion

In this large retrospective cohort of critically ill patients receiving dexmedetomidine, bradycardia occurred in nearly one in four cases (22.4%). Independent risk factors included moderate/severe liver disease, dementia, and cerebrovascular disease, while certain cardiovascular comorbidities—such as congestive heart failure and myocardial infarction—were associated with lower odds. Importantly, bradycardia was not independently associated with increased hospital mortality, and patients who developed bradycardia had slightly shorter ICU stays.

Our results challenge the widespread practice of avoiding or prematurely discontinuing dexmedetomidine because of bradycardia concerns, despite its proven ability to achieve the light sedation targets recommended by current guidelines.^5^ This paradox persists despite robust evidence that light sedation improves outcomes,^1,2,4,5^ and that dexmedetomidine is uniquely suited to achieve this goal.^1,2,4^ Cardiovascular concerns frequently push clinicians toward alternative sedatives such as propofol or benzodiazepines, which may undermine optimal sedation depth. The absence of an association between bradycardia and mortality (adjusted OR 0.99, p = 0.915) is consistent with prior systematic reviews showing no difference in mortality compared to other sedatives.12 The shorter ICU stays observed among patients with bradycardia (10.4 vs. 10.8 days, p = 0.012) suggest that bradycardia may serve as a physiologic marker of effective drug exposure, reflecting the benefits of dexmedetomidine’s sedation profile, including reduced delirium, earlier mobilization, and expedited liberation from mechanical ventilation.^1,4^

The risk factor analysis offers important clinical guidance. Patients with moderate/severe liver disease had the highest bradycardia risk (OR 1.87), likely due to impaired hepatic metabolism leading to higher circulating drug concentrations,^3^ underscoring the importance of careful titration rather than avoidance. The association between dementia and bradycardia may reflect altered central sensitivity and autonomic dysfunction in these patients.^10,11^ Conversely, patients with heart failure (OR 0.71) or myocardial infarction (OR 0.73) had lower odds of developing bradycardia, possibly due to dexmedetomidine’s sympatholytic action^6^ attenuating elevated baseline sympathetic tone, which may also reduce myocardial oxygen demand.^9^

From a clinical perspective, these findings provide important context for bradycardia management during dexmedetomidine therapy. While our study found no independent association between bradycardia and hospital mortality and observed shorter ICU length of stay in patients who developed bradycardia, these findings should be interpreted cautiously. The shorter ICU stays may reflect multiple factors including effective sedation management, and do not necessarily indicate that bradycardia itself is beneficial.^5,12^ These results are consistent with previous studies suggesting that dexmedetomidine-associated bradycardia may not translate to worse clinical outcomes when hemodynamic stability is maintained.^12^ For higher-risk patients, such as those with liver disease or dementia, enhanced monitoring remains appropriate. Clinical decisions regarding dexmedetomidine continuation should continue to be individualized, considering both heart rate changes and overall hemodynamic status, with further prospective research needed to establish optimal management protocols for bradycardia during dexmedetomidine therapy.

### Study Limitations

This study has several limitations. The retrospective design makes the study vulnerable to selection bias and unmeasured confounding factors. The identification of comorbidities relied on ICD coding, which introduces potential for coding inaccuracies. Due to the observational nature, definitive causal conclusions cannot be drawn. Lastly, the study was conducted within a single healthcare system, which may limit generalizability to other clinical settings.

## Conclusions

In this large cohort of critically ill patients, dexmedetomidine-associated bradycardia occurred in almost one-quarter of patients. Moderate/severe liver disease, dementia, and cerebrovascular disease were identified as significant risk factors, while certain cardiovascular comorbidities appeared protective. Importantly, bradycardia was not independently associated with increased hospital mortality. Future prospective studies should validate these identified risk factors and evaluate optimal management strategies for bradycardia during dexmedetomidine therapy.

## Supplementary Files

This is a list of supplementary files associated with this preprint. Click to download.

- BRAIDSupplementaryMaterials7.30.2025.docx

## Figures and Tables

**Table 1:** Baseline characteristics of patients with and without bradycardia

Characteristic	No Bradycardia (N = 3,963)	Bradycardia (N = 1,143)	P-value
**Demographics**			
Age (mean ± SD)	58.8 ± 15.5	61.6 ± 15.2	<0.001
Sex (Male)	2293 (57.9%)	613 (53.6%)	0.012
White	2289 (57.8%)	680 (59.5%)	
Black or African American	1283 (32.4%)	365 (31.9%)	
Asian	98 (2.5%)	33 (2.9%)	
Unknown	238 (6.0%)	54 (4.7%)	
Other	25 (0.6%)	8 (0.7%)	
Native Hawaiian or Other Pacific Islander	15 (0.4%)	2 (0.2%)	
American Indian or Alaska Native	15 (0.4%)	1 (0.1%)	
BMI (mean ± SD)	29.3 ± 8.8	29.9 ± 10.3	0.595
Comorbidities			
Myocardial Infarction	758 (19.1%)	206 (18.0%)	0.423
Congestive Heart Failure	2108 (53.2%)	576 (50.4%)	0.100
Peripheral Vascular Disease	1223 (30.9%)	287 (25.1%)	<0.001
Cerebrovascular Disease	1248 (31.5%)	392 (34.3%)	0.081
Dementia	169 (4.3%)	87 (7.6%)	<0.001
COPD	1428 (36.0%)	461 (40.3%)	0.009
Rheumatological Disease	201 (5.1%)	67 (5.9%)	0.328
Peptic Ulcer Disease	214 (5.4%)	85 (7.4%)	0.012
Diabetes	815 (20.6%)	187 (16.4%)	0.002
Hypertension	327 (8.3%)	104 (9.1%)	0.398
Renal Disease	1202 (30.3%)	357 (31.2%)	0.587
Cancer	404 (10.2%)	114 (10.0%)	0.869
Moderate/Severe Liver Disease	283 (7.1%)	115 (10.1%)	0.002
AIDS	85 (2.1%)	13 (1.1%)	0.027
Clinical Outcomes			
Mortality	406 (10.2%)	130 (11.4%)	0.297
SOFA Score (mean ± SD)	4.4 ± 3.1	4.7 ± 3.1	<0.001
Length of Stay (mean ± SD)	10.8 ± 10.6	10.4 ± 10.1	0.012

## Data Availability

The datasets used and/or analyzed during the current study are available from the corresponding author on reasonable request.

## Abbreviations

BMI: Body mass index
CI: Confidence interval
ICU: Intensive care unit
LOS: Length of stay
OR: Odds ratio
SOFA: Sequential Organ Failure Assessment
